# Estimated Glomerular Filtration Rate and Mortality among Patients with Coronary Heart Disease

**DOI:** 10.1371/journal.pone.0161599

**Published:** 2016-08-18

**Authors:** Qian Chen, Yuan Zhang, Ding Ding, Min Xia, Dan Li, Yunou Yang, Qing Li, Jiaxing Liu, Xuechen Chen, Gang Hu, Wenhua Ling

**Affiliations:** 1 Guangdong Provincial Key Laboratory of Food, Nutrition and Health, School of Public Health, Sun Yat-sen University, Guangzhou, Guangdong, China; 2 Chronic Disease Epidemiology Laboratory, Pennington Biomedical Research Center, Baton Rouge, LA, United States of America; 3 Department of Cardiology, General Hospital of Guangzhou Military Command of People’s Liberation Army, Guangzhou, Guangdong, China; University of Sao Paulo Medical School, BRAZIL

## Abstract

**Objective:**

The association between estimated glomerular filtration rate (eGFR) and the risk of mortality among patients with coronary heart disease (CHD) is complex and still unclear. The aim of this study was to evaluate the effect of eGFR on the risk prediction of all-cause and cardiovascular disease (CVD) mortality with a long follow-up period among patients with CHD in China.

**Methods:**

We conducted a prospective cohort study of 3276 Chinese patients with CHD. Cox proportional hazards regression models were used to estimate the association of different levels of eGFR with the risks of mortality.

**Results:**

During a mean follow-up period of 4.9 years, 293 deaths were identified. The multivariable-adjusted hazard ratios associated with different levels of eGFR (≥90 [reference group], 60–89, 30–59, 15–29 ml/min per 1.73m^2^) at baseline were 1.00, 1.28 (95% confidence interval [CI], 0.87–1.88), 1.96 (95% CI, 1.31–2.94), and 3.91 (95% CI, 2.15–7.13) (*P* <0.001) for all-cause mortality, and 1.00, 1.26 (95% CI, 0.78–2.04), 1.94 (95% CI, 1.17–3.20), and 3.77 (95% CI, 1.80–7.89) (*P* <0.001) for CVD mortality, respectively. After excluding subjects who died during the first 2 years of follow-up (n = 113), the graded associations of eGFR with the risks of all-cause and CVD morality were still present. The addition of eGFR to a model including traditional cardiovascular risk factors resulted in significant improvement in the prediction of all-cause and CVD mortality.

**Conclusions:**

Reduced eGFR (< 60 ml/min per 1.73 m^2^) at baseline is associated with increased risks of all-cause and CVD mortality among Chinese patients with CHD.

## Introduction

Cardiovascular diseases (CVD) and chronic kidney disease (CKD) are two important health problems [1–3]. CVD is the leading cause of deaths worldwide in 2012 (17.5 million deaths of the world’s 56 million deaths) [1]. Several previous studies have demonstrated that the major cause of death in non-dialysis-dependent CKD was CVD [4–6]. Meanwhile, some studies have indicated a significant association between severity of CKD assessed by estimated glomerular filtration rate (eGFR) and the risk of CVD in the general population and high-risk population [7–12]. The linkage between kidney dysfunction and CVD risk attracts lots of attention.

Previous studies have shown that coronary heart disease (CHD) patients with lower eGFR were at a higher risk of poor outcomes compared with those with normal eGFR (≥90 ml/min per 1.73m^2^) [13, 14]. It has been found that the prevalence of CHD and CKD continuously increase during the past two decades in China [15–17], however, very few studies have assessed the association between eGFR and the risks of all-cause or CVD mortality among Chinese people with CHD [18, 19]. Moreover, these studies in China have been limited by small sample size [18] and short-term follow-up [19], and the potential bias caused by premature death or the presence of occult diseases at baseline may be concealed. Clarifying long-term death risk associated with kidney function such as eGFR among patients with CHD is important for improving the clinical treatment and prognostic evaluation. We investigated the association between eGFR and the risks of all-cause and CVD mortality with a long follow-up period among Chinese patients with CHD.

## Materials and Methods

### Study Population

The Guangdong Coronary Artery Disease Cohort (GCADC) is a prospective, hospital-based cohort. Details of the GCADC study about aims, selection, criteria and ascertainment of CHD have been published previously [20, 21]. Using the same selection, criteria and ascertainment of CHD, we firstly recruited 1984 patients during 2008–2011, and then further included 1615 patients via electronic medical records during 2013–2014. Briefly, we recruited 3599 successive patients admitted to the Cardiology Department of three superior specialty hospitals in Guangdong, China (Guangzhou Military General Hospital, Sun Yat-Sen Memorial Hospital, and First Affiliated Hospital of Sun Yat-Sen University) between October 2008 and December 2011, and diagnosed as CHD according to World Health Organization 1999/2000 guidelines [22, 23]. The present analyses included 3276 CAD patients aged 40 years or older after excluding participants with incomplete data at baseline (n = 295), eGFR < 15 ml/min per 1.73m^2^ (n = 14), and clinically implausible estimate of kidney function (serum creatinine <0.28 mg/dl) at baseline (n = 14). Written informed consent was obtained from each of the first recruited patients from the GCADC study. We did not obtain informed consent from those additional participants involved in the present study because we used anonymized data compiled from electronic medical records. All participants of the study were conducted according to the principles expressed in the 1975 Declaration of Helsinki and the Sun Yat-Sen University Ethics Committee approved the study.

### Measurements

In the present study, patient’s general information was ascertained through a face-to-face interview described previously [20] or extracted from the hospital electronic system, including examination date, birth date, gender, address, education level, marriage, leisure-time physical activity, smoking habits, alcohol consumption, history of diabetes and hypertension, and use of medication before admission. Smoking habits and alcohol consumption have been divided into three groups: never, past, or current as described previously [21].

All participants underwent clinical assessment after hospital admission. Clinical measurements of each participant were extracted from the hospital electronic record system. At admission, patients’ height, weight and blood pressure were measured by trained nurses using a standard protocol [24]. Body mass index (BMI) was defined as the weight in kilograms divided by the square of height in meters. Participants fasted overnight for at least 12h before venous blood specimen was drawn in the next morning after hospital admission. Lipids and fasting glucose were determined by standard methods using the Hitachi Automatic Analyzer 7600–020 (Hitachi, Tokyo, Japan).

### Kidney function

Serum creatinine level was measured using an enzymatic method by the Hitachi Automatic Analyzer 7600–020 (Hitachi, Tokyo, Japan). We estimated GFR using the Modification of Diet in Renal Disease (MDRD) Study equation for standardized serum creatinine [25]: estimated GFR (eGFR) = 175× (standardized serum creatinine in mg/dl)^-1.154^ × age^-0.203^ × 0.742 (if female). Estimated GFR is reported in ml/minute per 1.73 m^2^ of body surface area [3].

### Prospective Follow-up

Follow-up information was obtained from the hospital medical system of re-hospitalization, telephone contacts with patients or their immediate family members, and death registration at the Guangdong Provincial Center for Disease Control and Prevention annually. The surveys were followed to the end of September 2015 or patient’s death if the date was prior to September 2015. We used ICD codes to identify the cause of death; and the ICD codes I00–I99 were classified as CVD deaths.

### Statistical analyses

Differences in risk factors between the eGFR groups were tested by the general linear model after adjustment for age for continuous variables and Chi-square analysis for categorical variables. Cox proportional hazards models were performed to determine the associations between baseline eGFR levels and the risks of all-cause and CVD mortality. eGFR was analyzed in the following three ways: (1) as a continuous variable, (2) as a four-category variable (eGFR ≥ 90 [reference group], 60–89, 30–59, and 15–29 ml/min/1.73 m^2^) based on CKD classification recommended by the National Kidney Foundation [3], and (3) as a binary variable using the recommended cutoff of 60 ml/min per 1.73 m^2^ [26]. All analyses were adjusted for age, sex, education, smoking, alcohol drinking, marriage, leisure-time physical activity, fasting glucose, BMI, systolic blood pressure (SBP), high-density lipoprotein cholesterol (HDL-C), triglyceride, types of CHD, use of antihypertensive medications, use of glucose-lowering medications, use of lipid-lowering medications, and use of antiplatelet medications. To avoid the potential bias caused by premature death or the presence of occult diseases at baseline, we conducted additional analyses after excluding deaths occurring in the first 2 years of follow-up (n = 113). We also computed the C statistic associated with the risk-estimation model based on the major traditional CVD risk factors and the C statistic associated with the model based on a combination of the major traditional CVD risk factors and eGFR [27]. The discriminative ability of the models including and excluding eGFR was tested with the use of C statistic improvement [28]. Statistical significance was considered to be *P*<0.05. All statistical analyses were performed using PASW for Windows, version 20.0 (IBM SPSS Inc., Chicago, IL) and R for Windows, version 3.0.1.

## Results

General characteristics of the study population at baseline according to eGFR categories were presented in Table 1. After adjustment for age, eGFR had an inverse association with BMI, SBP, diastolic blood pressure, triglycerides, total cholesterol, history of diabetes, history of hypertension, using glucose-lowering medications and using antihypertensive medications, and a direct association with current alcohol drinking and current smoking.

**Table 1:** Baseline characteristics according to different levels of eGFR among patients with coronary heart disease.

Characteristics	eGFR, ml/min per 1.73m^2^				*P* value
	≥90	60–89	30–59	15–29	
No. of participants, %	705 (21.5)	1661 (50.7)	854 (26.1)	56 (1.7)	
Male, %	66.5	66.0	54.4	57.1	<0.001
Age, y	58.2 (0.40)	64.1 (0.26)	68.3 (0.36)	70.7 (1.42)	<0.001
Body mass index, kg/m^2^	23.7 (0.13)	23.9 (0.08)	24.4 (0.12)	24.7 (0.45)	0.001
Systolic blood pressure, mmHg	135 (0.85)	135 (0.54)	136 (0.76)	148 (2.92)	<0.001
Diastolic blood pressure, mmHg	76.9 (0.49)	78.2 (0.31)	78.6 (0.44)	81.4 (1.69)	0.012
Low-density lipoprotein cholesterol, mmol/L	2.96 (0.04)	2.97 (0.02)	3.03 (0.03)	3.17 (0.13)	0.20
High-density lipoprotein cholesterol, mmol/L	1.14 (0.01)	1.14 (0.01)	1.16 (0.01)	0.95 (0.04)	<0.001
Triglycerides, mmol/L	1.80 (0.06)	1.83 (0.04)	1.89 (0.05)	2.73 (0.19)	<0.001
Total cholesterol, mmol/L	4.74 (0.04)	4.76 (0.03)	4.89 (0.04)	5.07 (0.15)	0.007
Fasting glucose, mmol/L	7.11 (0.11)	6.49 (0.07)	6.30 (0.10)	6.88 (0.38)	<0.001
Types of coronary artery disease, %					0.61
Acute coronary syndrome	58.4	57.4	55.4	60.7	
Chronic coronary artery disease	41.6	42.9	44.6	39.9	
Married, %	71.3	77.8	82.1	73.2	<0.001
Years of education, %					0.001
≤9	65.6	64.7	74.1	76.2	
10–12	17.4	18.0	10.7	11.9	
≥13	17.0	17.3	15.2	11.9	
Smoking, %					<0.001
Never	56.1	60.6	66.8	70.9	
Past	7.0	10.2	10.4	12.7	
Current	36.9	29.2	22.8	16.4	
Alcohol drinking, %					0.001
Never	78.4	84.5	87.1	87.5	
Past	5.4	4.2	3.4	4.2	
Current	16.2	11.2	9.5	8.3	
Leisure-time physical activity, %					0.51
None	35.0	32.0	33.3	43.5	
<30 minutes/day	23.4	20.3	21.0	17.4	
≥30 minutes/day	41.6	47.7	45.7	39.1	
History of hypertension, %	76.7	74.5	80.0	94.6	<0.001
History of diabetes, %	21.8	21.2	26.7	44.6	<0.001
History of dyslipidemia, %	24.8	18.6	16.3	14.3	<0.001
Uses of medications before admission,%					
Antihypertensive medication	40.0	48.6	60.8	65.5	<0.001
Glucose-lowering medication	14.9	15.4	20.1	29.1	0.001
Lipid-lowering medication	10.6	10.8	11.5	8.2	0.87
Antiplatelet medication	16.2	15.0	16.3	18.4	0.79

The values are mean (SE) or percentage.

Abbreviation: eGFR, estimated glomerular filtration rate.

During a mean follow-up of 4.9 years, there were 293 deaths recorded, and 189 of these were due to CVD. The multivariable-adjusted (age, sex, education, smoking, alcohol drinking, marriage, leisure-time physical activity, fasting glucose, BMI, SBP, HDL-C, triglyceride, types of CHD, use of antihypertensive medications, use of glucose-lowering medications, use of lipid-lowering medications, and use of antiplatelet medications) hazard ratios associated with different levels of eGFR (≥ 90, 60–89, 30–59, and 15–29 ml/min/1.73 m^2^) at baseline were 1.00, 1.28 (95% confidence interval [CI], 0.87–1.88), 1.96 (95% CI, 1.31–2.94), and 3.91 (95% CI, 2.15–7.13) (*P* for trend <0.001) for all-cause mortality, and 1.00, 1.26 (95% CI, 0.78–2.04), 1.94 (95% CI, 1.17–3.20), and 3.77 (95% CI, 1.80–7.89) (*P* for trend <0.001) for CVD mortality, respectively (Table 2).

**Table 2:** HRs (95% CI) of all-cause and cardiovascular disease mortality according to different levels of eGFR by different follow-up periods among patients with coronary heart disease.

eGFR, ml/min per 1.73m^2^	No. of deaths			HR (95% CI) ^a^	
	Total	CVD	Peron-years	All-cause mortality	CVD mortality
All participants					
≥90	34	22	3552	1.00	1.00
60–89	129	83	8153	1.28 (0.87–1.88)	1.26 (0.78–2.04)
30–59	112	72	4151	1.96 (1.31–2.94)	1.94 (1.17–3.20)
15–29	18	12	246	3.91 (2.15–7.13)	3.77 (1.80–7.89)
P for trend				<0.001	<0.001
After excluding patients died during the first 2-year of follow up					
≥90	18	12	3537	1.00	1.00
60–89	80	44	8119	1.46 (0.87–2.45)	1.18 (0.62–2.27)
30–59	70	43	4115	2.16 (1.26–3.72)	2.00 (1.02–3.93)
15–29	12	7	242	5.06 (2.35–10.9)	4.41 (1.66–11.7)
P for trend				<0.001	0.003
First 2-year of follow-up					
≥90	16	10	1393	1.00	1.00
60–89	49	39	3258	1.10 (0.62–1.97)	1.37 (0.67–2.79)
30–59	42	29	1659	1.75 (0.95–3.23)	1.91 (0.89–4.08)
15–29	6	5	105	3.21 (1.19–8.61)	3.80 (1.23–11.8)
P for trend				0.024	0.077

Abbreviations: HR, hazard ratio; CI, confidence interval; CVD, cardiovascular disease; and eGFR, estimated glomerular filtration rate.

^a^ Adjusted for age, sex, education, smoking, marriage, alcohol drinking, marriage, leisure-time physical activity, fasting glucose, body mass index, systolic blood pressure, high-density lipoprotein cholesterol, triglycerides, types of coronary artery disease, use of antihypertensive medications, use of glucose-lowering medications, use of lipid-lowering medications, and use of antiplatelet medications.

After excluding subjects who died during the first 2 years of follow-up (n = 113), the multivariable-adjusted hazard ratios associated with different levels of baseline eGFR (≥ 90, 60–89, 30–59, and 15–29 ml/min/1.73 m^2^) were 1.00, 1.46 (95% CI, 0.87–2.45), 2.16 (95% CI, 1.26–3.72), and 5.06 (95% CI, 2.35–10.9) (*P* for trend <0.001) for all-cause mortality, and 1.00, 1.18 (95% CI, 0.62–2.27), 2.00 (95% CI, 1.02–3.93), and 4.41 (95% CI, 1.66–11.7) (*P* for trend = 0.003) for CVD mortality, respectively. When we conducted an additional analysis on the first 2 years of follow-up, the multivariate-adjusted hazard ratios associated with different levels of baseline eGFR (≥ 90, 60–89, 30–59, and 15–29 ml/min/1.73 m^2^) were 1.00, 1.10 (95% CI, 0.62–1.97), 1.75 (95% CI, 0.95–3.23), and 3.21 (95% CI, 1.19–8.61) (*P* for trend = 0.024) for all-cause mortality, and 1.00, 1.37 (95% CI, 0.67–2.79), 1.91 (95% CI, 0.89–4.08), and 3.80 (95% CI, 1.23–11.8) (*P* for trend = 0.077) for CVD mortality, respectively (Table 2).

When stratified by sex, age, history of diabetes, types of CAD, use of glucose-lowering drugs, and use of antihypertensive drugs, the inverse association between eGFR and the risks of all-cause and CVD mortality was still significant in most of subgroups. There was no interaction of eGFR and sex, age, types of CAD, and use of antihypertensive drugs on the risks of all-cause and CVD mortality (all *P* >0.05). The interaction of eGFR and history of diabetes and use of glucose-lowering medications was significant on the risk of CVD mortality (all *P* <0.05), but not all-cause mortality (all *P* >0.05) (Table 3).

**Table 3:** HRs (95% CI) ^a^ of all-cause and cardiovascular disease mortality according to different levels of eGFR among patients with coronary heart disease of various subpopulations.

	All-cause mortality			CVD mortality		
	eGFR ≥60	eGFR <60	*P* for interaction	eGFR ≥60	eGFR <60	*P* for interaction
	ml/min per 1.73m^2^	ml/min per 1.73m^2^		ml/min per 1.73m^2^	ml/min per 1.73m^2^	
Gender			>0.50			>0.75
Male	1.00	1.73 (1.27–2.35)		1.00	1.82 (1.26–2.63)	
Female	1.00	1.72 (1.13–2.62)		1.00	1.57 (0.88–2.77)	
Age groups, y			>0.75			>0.75
<65	1.00	1.99 (1.09–3.63)		1.00	1.88 (0.89–4.00)	
≥65	1.00	1.92 (1.47–2.50)		1.00	1.97 (1.42–2.74)	
History of diabetes			>0.10			<0.05
No	1.00	1.57 (1.15–2.15)		1.00	1.50 (1.01–2.24)	
Yes	1.00	2.29 (1.50–3.49)		1.00	2.33 (1.40–3.86)	
Types of coronary artery disease			>0.25			>0.05
Chronic coronary artery disease	1.00	1.89 (1.26–2.85)		1.00	2.04 (1.18–3.51)	
Acute coronary syndrome	1.00	1.61 (1.18–2.19)		1.00	1.63 (1.12–2.37)	
Use of glucose-lowering medications			>0.10			<0.05
No	1.00	1.58 (1.19–2.10)		1.00	1.48 (1.04–2.12)	
Yes	1.00	2.87 (1.65–4.97)		1.00	3.30 (1.63–6.68)	
Use of antihypertensive medicines			>0.05			>0.25
No	1.00	1.38 (0.93–2.05)		1.00	1.30 (0.79–2.15)	
Yes	1.00	2.07 (1.49–2.88)		1.00	2.13 (1.42–3.20)	

Abbreviation: HR, hazard ratio; CI, confidence interval; CVD, cardiovascular disease; and eGFR, estimated glomerular filtration rate.

^a^ Adjusted for age, sex, education, smoking, marriage, alcohol drinking, marriage, leisure-time physical activity, fasting glucose, body mass index, systolic blood pressure, high-density lipoprotein cholesterol, triglycerides, types of coronary artery disease, use of antihypertensive medications, use of glucose-lowering medications, use of lipid-lowering medications, and use of antiplatelet medications, other than the variable for stratification.

The C statistic was used to assess and quantify the improvement in risk prediction for all-cause and CVD mortality offered by eGFR levels. Table 4 showed that the addition of eGFR to the fully adjusted model increased the C-index from 0.761 to 0.767 (P <0.001) for all-cause mortality, and from 0.772 to 0.778 (P <0.001) for CVD mortality, respectively.

**Table 4:** Impact of eGFR levels and metrics of discrimination.

	All-cause mortality		Cardiovascular disease mortality	
	Clinical model ^a^	Clinical model + eGFR	Clinical model ^a^	Clinical model + eGFR
C Index	0.761	0.767	0.772	0.778
*P*		<0.001		<0.001

Abbreviations: eGFR, Glomerular filtration rate.

^a^ Clinical model including age, sex, education, smoking, marriage, alcohol drinking, marriage, leisure-time physical activity, fasting glucose, body mass index, systolic blood pressure, high-density lipoprotein cholesterol, triglycerides, types of coronary artery disease, use of antihypertensive medications, use of glucose-lowering medications, use of lipid-lowering medications, and use of antiplatelet medications.

## Discussion

This study demonstrated that baseline reduced eGFR (< 60 ml/min per 1.73 m^2^) was associated with increased risks of all-cause and cardiovascular mortality among Chinese patients with CHD.

One large community-based study of 1,120,295 adults observed an independent graded association between a decreased eGFR and the risks of death as well as CVD events [29]. Although previous studies have found an association between a decreased eGFR and CVD risk, most of these studies were from the western general population [4, 8], or other high-risk population [9,30]. Zhang et al. [31] conducted an observational prospective study in rural areas of China, and found an inverse association between eGFR and all-cause and cardiovascular mortality among patients with hypertension. Several studies have already observed an increased risk of poor outcomes with a decreased eGFR among patients with prior CVD. In a nationally representative cohort of elderly Medicare patients with acute myocardial infarction (AMI), patients with eGFR (30–60, and < 30 ml/min per 1.73 m^2^) showed a 21% and 77% higher risk of 10-year mortality compared with those with eGFR ≥60 ml/min per 1.73 m^2^ [32]. In the Anemia in Chronic Heart Failure: Outcomes and Resource Utilization (ANCHOR) Study, an increased death risk was found among patients with eGFR <60 ml/min per 1.73 m^2^ compared with those with eGFR ≥60 ml/min per 1.73 m^2^ [11]. Participants enrolled in these studies, however, were considered having a severe CHD status. In the present study, we included CHD patients with both acute coronary syndrome (ACS) and chronic manifestation who may have both severe and stable status. We found an inverse association between reduce eGFR (<60 ml/min per 1.73 m^2^) and the risks of all-cause and CVD mortality among Chinese patients with both ACS and chronic coronary artery disease, and with acute and chronic status combined. This gives a very important message for clinical treatment for CHD patients.

In the present study, about 38.6% of deaths occur within the first 2 years of follow-up, it is possible that some biomarkers of severity of the acute CAD events would dominate the explanation of short-term mortality risks. Our study is the first to demonstrate the association between eGFR and mortality within the first 2 years of follow-up especially after excluding CHD patients who died during the first 2 years of follow-up. We found that the inverse association between eGFR and mortality was consistent when the follow-up was limited to the first 2 years or we excluded CHD patients who died during the first 2 years of follow-up.

The reasons for the increased risk of all-cause and CVD mortality in CHD patients with a decreased eGFR are not fully elucidated. Several possible mechanisms exist. It has been shown that people with impaired kidney function have a high prevalence of traditional risk factors or even nontraditional risk factors for CVD, such as older age, diabetes, hypertension, dyslipidemia, elevated fibrinogen and low serum albumin [26, 33], and the association between these risk factors and the risks of all-cause and CVD mortality was well established [20, 34–36]. However, in the present study, the linkage between eGFR and the risks of all-cause or CVD mortality was independent from major CVD risk factors. Thus, impaired kidney function may affect the prognosis of CHD patients through a variety of mechanisms. Kidney dysfunction may accelerate the atherosclerosis. Manjunath et al. demonstrated that low level of eGFR was an independent risk factor for atherosclerotic CVD [37]. Inflammation [26] and oxidative stress [37] may partly be responsible for the association [36]. Furthermore, decreased eGFR may reflect kidney damage like the loss of nephrons and kidney fibrosis or lower clearance that leads to increased mortality risk through accumulation of uremic toxins, higher plasma levels of CVD risk markers like homocysteine and uric acid, and multiple metabolic abnormalities [8, 38]. Recently, increased promoters of calcification and reduced inhibitors of calcification were also used to explain the association between kidney insufficiency and CVD risk [36, 39]. On the other hand, cardiac insufficiency could significantly impair the renal function, which would further affect the prognosis of CHD patients. Interactions between heart and kidney are multiple and complex; primary dysfunction of one of heart and kidney often results in secondary injury to another one, and this phenomenon is called cardiorenal syndrome (CRS) [40]. Acute kidney injury (AKI) plays a harmful role in prognosis among ACS patients [33]. Bruetto et al. [41] found that reduced eGFR (< 60 ml/min/1.73 m^2^) with and without AKI was associated with an increased risk of 30-day mortality, but the association of impaired admission eGFR without AKI with an increased 30-day to 1-year mortality hazard was not significant among patients with acute myocardial infarction. However, most of our enrolled patients had only serum creatinine measured once at admission, and repeated serum creatinine data during hospital stay and urine output were unavailable in the present study. Thus we could not estimate the effect of AKI and reduced eGFR on the risk of later mortality in the present study.

In order to improve the prognosis of CHD patients, we need a more valuable prediction method integrating multiple valuable indicators in the clinic. Our results further indicate that eGFR significantly improves risk prediction for both all-cause and CVD mortality. This finding emphasizes the need for treatments to slower the decreasing speed of eGFR among CHD patients.

There are several limitations in this study. First, serum creatinine concentration was measured only once at baseline and urine output data were unavailable. Thus we could not estimate the joint effect of AKI and eGFR on the risk of morality among patients with CHD. Second, we enrolled participants from hospitals where in-patients may have a more severe disease status. In order to attenuate this bias, we recruited both ACS patients and chronic CHD patients, and some of these participants were electively admitted patients with mild status. Finally, clinical indexes were detected based on each patient’s condition and each physician’s discretion, and we were unable to obtain some risk factors for CVD [36].

In conclusion, we found that reduced eGFR at baseline was associated with increased risks of all-cause and cardiovascular mortality among CHD patients in China.
